# Evaluating the impact of a laboratory teaching innovation: the case of the Bioskills at home kit

**DOI:** 10.1099/acmi.0.001157.v3

**Published:** 2026-05-21

**Authors:** Bunmi B. Omorotionmwan, Sarah Rayment, Karin Garrie, Ishwinder Kaur, Gareth McVicker, Jody Winter, David Negus

**Affiliations:** 1 Department of Biosciences, School of Science and Technology, Nottingham Trent University, Clifton Lane, Nottingham NG11 8NS, UK

**Keywords:** employability, home learning, laboratory skills, meaningful learning, personal tutorial, pipetting, practical skills, quality education

## Abstract

Practical laboratory training is a key component of biosciences education, essential for developing critical skills and bridging the gap between theory and practice. The ‘Bioskills at home kit’, originally developed during the COVID-19 pandemic, was created to support first-year students in developing core laboratory skills at a time when access to in-person sessions was limited. Each kit contained equipment and guided activities focused on pipetting, microscopy, experimental design and microbial growth curve analysis. This study aimed to assess the educational impact of this kit using a mixed-methods approach. Data were collected across three student cohorts (2020/21, 2021/22 and 2024/25) using surveys and a focus group and analysed using appropriate statistical analyses and reflexive thematic analysis, respectively. The study evaluated the impact of the Bioskills at home kit on student learning across the cognitive, affective and psychomotor domains. Aspects of student experience, including perceived benefits, barriers to engagement and suggestions for improvement, were investigated, and student attainment from a compulsory laboratory assessment spanning seven academic years (2018/19–2024/25) was analysed. Results show that students experienced improved understanding of theoretical concepts, confidence, enjoyment and enhanced technical skills. Student attainment in practical assessment was also improved. Embedding the delivery of the kit in a more structured way within the personal tutorial system in subsequent years improved student engagement which was a challenge during the first year of intervention. Here, we demonstrate the successful implementation of the Bioskills at home kit which offers a scalable and inclusive model for flexible laboratory teaching.

## Data Summary

The authors confirm that additional supporting data have been made available through the supplementary data files and on Figshare: https://doi.org/10.6084/m9.figshare.28847567.v1.

## Introduction

Laboratory classes play a central role in biosciences education [1] providing numerous benefits to students [2,3]. Such environments create opportunities for students to develop practical skills, as well as experimental design and data analysis, foundational for academic progress and future career success. Additionally, laboratory sessions create stimulating spaces for students to experience theoretical concepts in a concrete way, promoting understanding of complex bioscience ideas. Particularly for first-year undergraduate students, the development of core technical skills such as accurate pipetting, microscopy and serial dilution is critical for success throughout their university journey, and there is a consensus that proficiency in these basic lab skills is a key aspect of the undergraduate curriculum [4,6]. Besides, based on the QAA Benchmark standards, laboratory skills development is an accreditation requirement of professional bodies, like the Royal Society of Biology [7].

### Experiential learning and meaningful learning in science education

#### Experiential learning

Based on Dewey’s constructivism theory of experience that education is to be conceived as a continuing reconstruction of experience, the Experiential Theory of learning has been defined as ‘the process whereby knowledge is created through the transformation of experience and knowledge results from the combination of grasping and transforming experience’ [8,9]. According to Kolb’s model of experiential learning, knowledge is being constructed when students engage with the stages of concrete experience, abstract conceptualization, reflective observation and active experimentation, creating a learning cycle of grasping and transforming experience [9].

Within the bioscience laboratory, students are assigned practical tasks requiring hands-on learning and collaboration, promoting understanding and construction of knowledge through experience. These align with central themes of experiential learning such as active learning and social engagement [10,11]. Experiential learning has been shown to have benefits on students' learning. An increase in the technical skills, confidence, student outcome and engagement in first-year undergraduate bioscience students was reported following lab sessions which allowed experiential learning situated within an authentic context [12].

#### Meaningful learning

In a laboratory setting, there is ongoing active learning. This enables students to interact with their experiences, make connections with abstract theories and, within that context, construct meaning. According to Novak [13], ‘meaningful learning occurs when new knowledge is consciously linked by the learner to existing concepts or propositions the learner already knows’. This is usually based on the combination of cognitive, affective and psychomotor domains [13].

It is based on this learning theory of Novak [13] that Galloway and Bretz [14] designed the Meaningful Learning in the Laboratory Instrument as a model/tool to evaluate student learning in the undergraduate chemistry laboratory. In their work, they evidence that in the laboratory, students’ ‘doing’ patterns are based on how they think and feel [15]. They propose that for meaningful learning to occur within the laboratory, students should be able to merge their thoughts and feelings (expectations) with what they are actively doing (experience). When meaningful learning through active hands-on activities takes place in the laboratory, students can make connections with prior knowledge and consolidate understanding beyond rote learning.

### Challenges with traditional laboratory provision and the need for innovative teaching

Despite the many benefits of laboratory sessions, traditional laboratory provision has faced significant challenges over the years, including limited access to facilities and resources, further amplified by the COVID pandemic, occasioning disruptions to face-to-face laboratory sessions [16]. This shift highlighted the need for innovative and flexible approaches in the delivery of practical sessions to students, beyond on-campus provision. A range of alternatives to in-person laboratory sessions has been explored, and one such example is the online delivery of practical sessions. Goldberg and Dintzis [17] describe the delivery of virtual microscopy labs with team-based learning to first-year physiology/histology students at the Johns Hopkins University which supported an improvement in assessment outcome. In addition, using a blended approach, the Dynamic Laboratory Manual has been reported to boost student level of preparation and confidence in Bristol. This e-learning tool, comprising interactive pre-lab videos, lab simulations and assessments, enabled students to make the best use of laboratory time and engage in higher order learning [18, 19].

While the adoption of remote/online learning models has its benefits, complications in this delivery can arise based on the disparity of student access to basic technology and internet resources which was revealed during the COVID pandemic [20]. Such digital divides can create inequalities and disadvantages to students from less privileged backgrounds [21]. For instance, 1 out of 10 students interviewed noted that limited access to a personal computing device required them to perform most learning tasks on a mobile phone, as laptops and desktops were shared among family members [3]. Moreover, the dynamics of higher education are evolving with an increasing need for more inclusive teaching practices to ensure access and participation for all students. Ensuring that disadvantaged students have equal opportunities to succeed therefore requires more flexible innovations in our teaching design and delivery. This will help future-proof our education provision and enhance both student satisfaction and outcomes.

A valuable alternative to traditional labs in situations of limited access is virtual lab simulations. They not only facilitate self-directed learning for students and enhance confidence prior to physical lab sessions but are also cost-efficient to universities with reference to provision of practical resources and lab consumables [22]. Virtual labs can complement in-person lab sessions and have been shown to enhance confidence and skill development prior to entering the physical laboratories [23,24]. While virtual labs have their advantages, a drawback however is that they do not provide the actual hands-on training for techniques, and there is no physical environment for psychomotor experience. They also do not fully provide the opportunity to practise the real-world troubleshooting and critical thinking associated with lab classes. Physical contact appears to be a key aspect of experiential learning [10], which is missing from simulated labs.

### The Bioskills at home kit

The Bioskills at home kit was developed in response to the pandemic to support first-year bioscience students at Nottingham Trent University (NTU) in developing core laboratory skills at a time when there was limited access to the laboratory [25]. The kits contained equipment and activities in microscopy, pipetting, microbial growth curve, data handling and experimental design. The first year of intervention with the Bioskills at home kit was 2020/21 with refinements in subsequent years.

#### Initial roll-out of kit

The first iteration of this kit was delivered to all first-year students (466) in the Biosciences department in the 2020/21 academic session. The kit contained a digital microscope, micropipettes, safety glasses, dried yeast, haemocytometer slides and resources to carry out activities on microscopy, pipetting, microbial growth curve and haemocytometry. The resource list has been described and is available [26]. Students were required to carry out the kit activities at home following the guidance provided in the NTU online learning room, including illustrative videos and a discussion board for students to post questions they had with the kit. Participation was further encouraged through competitions such as the microscopy competition.

With the Bioskills at home kit intervention during the COVID-19 pandemic, it was possible to give students hands-on lab experience remotely, support the development of core technical skills at home and facilitate some community building among first-year students [25].

#### Associated compulsory assessment

The *Practical Techniques* assessment is mandatory for all first-year students in the Bioscience department at NTU. This practical laboratory test assessed competency in accurate pipetting and dilutions. It was conducted through live observation, and students received either a pass or fail grade. During the 2020/21 session, the Bioskills at home kit supported the maintenance of similar levels of success, as with other years, in the compulsory *Practical Techniques* assessment [25].

Despite the benefits of the Bioskills at home kit, there were challenges that limited its implementation, the most significant being the lack of student engagement with some aspects of the kit. For example, it was observed that 22.9% of students engaged with the online guidance for the pipetting home activity, while 12.7–19.6% of students watched the first exercise video on how to use the pipette [25]. These analytics of engagement with the supporting online resources were generally lower than those for other formative activities such as pre-recorded lectures, seminars and laboratory protocols [25]. The lack of definite structure such as clear deadlines for associated tasks may have contributed to the lack of commitment from the students.

#### Subsequent kit interventions

In order to improve student engagement in subsequent years (2021/22 onwards), the Bioskills at home kit was embedded within the personal tutorial system. At NTU, the personal tutorial system provides both pastoral care and academic support for all students and, in some cases, contributes to the delivery and assessment of key coursework. The tutorials are standardized across the department, and tutors are trained to deliver content with guidance sheets provided per session. This created a more structured delivery of kit activities plus a defined space and time to practise with tutor support before students took the kits home for continued practice. The contents of the kit were also streamlined to contain activities of the highest value to make for a more economical provision [26].

This study aimed to evaluate the impact of the Bioskills at home teaching innovation on students’ learning experience and outcome. We sought to investigate the initial barriers to engagement with the kit and how subsequent changes such as enhanced support through the tutorial system affected engagement and attainment.

## Methods

The participants for this study were students at the Biosciences department, NTU, who had received the Bioskills at home kit during their first year. To investigate the impact of the Bioskills at home kit, data from three student cohorts were collected: 2020/21 (this was the first cohort to receive the kit and was during the COVID pandemic, with little or no access to laboratory sessions), 2021/22 (representing the second cohort to receive the kit, embedded within the tutorial system, post-pandemic with some limitations to laboratory sessions) and 2024/25 (representing the fifth cohort to receive the kit, embedded within the tutorial system, post-pandemic, with full access to all laboratory sessions). Data were collected using focus groups and surveys. In addition, student outcomes in the associated compulsory *Practical Techniques* module assessment were analysed pre- and post-intervention, covering academic sessions between 2018/19 and 2024/25. Attendance at tutorial sessions was also collected.

### Focus group

For the cohort which received the first iteration of the kit during the COVID-19 pandemic (2020/21), a focus group was used to allow in-depth exploration of students’ expectations and experiences and to enable follow-up on open-ended questions [27]. This student cohort was now in their final year, having returned to campus after the lockdown. Participants were invited to take part in the focus group through emails sent out to the cohort, and participation was incentivized. The number of students who volunteered to participate in the focus group was four (*n*=4). The focus group was not anonymous, was conducted face to face on campus and aimed to assess how the kit had supported their journey through university. The goals of the focus group were to explore students’ differing experiences of engaging with the NTU Bioskills at home kit, to better understand possible barriers to engagement with the kit so that these could be removed in future versions and to investigate how the kit and its delivery could be improved in future, including ease of use, enjoyability, motivation, perceived benefit and available support. Questions around how students experienced particular tasks in the kit were also explored. Analysis of data was done using reflexive thematic analysis according to Braun and Clarke’s approach [28]. There was no conflict of interest with facilitators of the focus group, and students understood that it would not have any impact on their grades. The focus group question list, participant information sheet and consent form have been made available in the Material S1, available in the online Supplementary Material.

### Online survey

Data for the 2021/22 and 2024/25 cohorts were collected using online surveys to allow a broader exploration of student experience [29]. This survey was hosted on the Jisc Online Surveys platform, and students had the opportunity to complete this at the end of their first year within a specified time frame, and participation was incentivized. The number of participants in the 2021/22 feedback survey was 62 (17%), while there were 28 (10%) responses in the 2024/25 academic session. This was a voluntary survey, and students were made aware that participation had no impact on their studies. Participation was anonymous. The survey explored the following areas – student knowledge and understanding, ease of use and overall experience, technical skills gained, perceived benefits and challenges faced. A five-point Likert scale questionnaire required students to rate statements either as strongly disagree, disagree, undecided, agree or strongly agree. Descriptive statistical analysis and Spearman correlation tests were done using GraphPad Prism [30]. Spearman correlation analysis was carried out on the 2024/25 data to determine the correlation between variables. Results from the five-point Likert scale were simplified to three points by combining ‘strongly agree’ with ‘agree’ and ‘strongly disagree’ with ‘disagree’ for this analysis. Survey questions, participant information and consent sheet are provided in Material S1.

### Student outcome

Every first-year bioscience student at NTU is required to undertake and pass the compulsory pipetting assessment within the *Practical Techniques* module. As pipetting was a core activity in the Bioskills at home kit, we sought to assess how the kit had impacted on the students’ assessment outcome. The pipettes in the kit were mostly the same as those used in the lab. Grades were awarded as either pass or fail, and results of students who passed at first attempt were analysed for seven academic sessions, between 2018/19 and 2024/25, spanning pre- and post-intervention with the kit. A Chi-square statistical test was performed using GraphPad Prism [30].

### Kit refinement

Following its initial rollout in 2020/21, the Bioskills at home kit has undergone iterative refinements in content and implementation. In 2021/22, the contents of the kit were streamlined to activities around pipetting and microbial growth curve, and its delivery was implemented through the personal tutorial system onwards. Full details of the original and streamlined kit contents and protocol are given in Material S2. After 2021/22, a return back policy for the kit was initiated to foster sustainable delivery. Table 1 summarizes the kit refinement across the years, including cohort sizes and kit cost.

**Table 1.** Summary of the Bioskills at home kit refinement

Year	Cohort size	Kit contents – detailed list available [26]	Embedded within tutorial?	Return back policy?	Cost per kit
**2020/21**	466	Digital microscope, micropipettes (2–20, 20–200 and 200–1,000 µl), safety glasses, tweezers, dried yeast, haemocytometer slides and associated consumables	No	No	£160
**2021/22**	420	Micropipette (20–200 µl), dried yeast and associated consumables	Yes	No	£44
**2024/25**	329	Micropipette (20–200 µl), dried yeast and associated consumables	Yes	Yes	£44

### Student engagement data

Student engagement data for the 2021/22 and 2024/25 academic sessions, following the embedding of the Bioskills at home kit within the tutorial system, were collected using attendance data at the tutorial sessions designed for the delivery of the kit activities. Average attendance across both years was calculated.

### Ethics

Ethics approval for all data collection was obtained from the Nottingham Trent University School of Science and Technology Non-invasive ethics committee (Ref: 1622760). Participants received participant information sheets, and consent was obtained in every case.

## Results

### Focus group data from 2020/21 student cohort

Focus group data collected from the first cohort that received the Bioskills at home kit were analysed using NVivo, V14 [31]. Structural codes were made to encompass these four areas of inquiry – student experience, benefits, barriers to engagement and future improvements. Table 2 shows the codes and references with the frequency of occurrence given in parenthesis.

**Table 2.** Structural codes and references used for analysis of focus group data with frequency of occurrence given in parenthesis

*Experience* *(12)*	*Benefits* *(12)*	*Barriers* *(19)*	*Improvement* *(18)*
Enjoyed the celebratory event (4)	Relevance to *Practical Technique* test (4)	Technical challenges with kit tasks (12)	Need for introductory video guidance (8)
Engaging microscope activity (3)	Connection to module-taught material and assessment (4)	Fear of getting it wrong at discussion boards (4)	Introduce scheduled dedicated time for activities (5)
Microscopy competition was fun (3)	Opportunity to practise skills (4)	Lack of interest (2)	Streamline kit contents/sustainability issues (3)
Interest in pipetting exercise (2)		Kit was hard to use (1)	Smaller subject-specific discussion boards to encourage engagement (2)

As shown in Table 2, data from the focus group indicated that participants had a positive experience engaging with the Bioskills at home kit. Students particularly enjoyed the competitive and celebratory elements of the microscopy activity. One participant remarked:

‘*I particularly liked the photo competition with the microscopy experiment, I thought that was really, really fun and enjoyed it*.’

In addition, participants found the pipetting activity both engaging and relevant to the compulsory assessment in the *Practical Techniques* module, noting that it enhanced their confidence and preparedness for this test. As one student explained:

‘*The pipetting activity was helpful for practising for the Practical Techniques test, and it was quite obvious to tell the people who practised from those who did not*.’

Overall, participants found the kit valuable for practising lab skills at home.

Regarding the barriers to engagement, the most commonly reported issues were related to technical challenges with the kit, including a lack of understanding of what to do and where to find support for the kit activities. For example, a student described some difficulties regarding the microbial growth curve experiment:

‘*I tried that one about 2-3 times, but the yeast did not grow each time. It didn’t work for some others on multiple occasions*.’

Participants expressed reluctance in engaging with the discussion boards, mentioning that they did not feel confident asking questions for fear of getting it wrong as quoted below:

‘*It was intimidating that year. We hadn't really gotten used to faces and names, and most people were chatting as ‘anonymous’. It was uncomfortable, and I didn’t want to be judged by students for asking an obvious question or told off by the lecturers*.’

Some others noted a general case of lack of interest as a barrier to engagement.

Following on from the barriers identified, participants proposed possible improvements to the kit and its implementation. These included providing clear introductory videos and dedicated time for guided practice with lecturers, and they noted the benefits of having smaller, subject-specific discussion boards to foster a better sense of community. Recommendations around sustainability to streamline and reuse some kit content in future iterations were also given.

### Survey data collected from 2021/22 and 2024/25 cohorts

Results for survey questions have been grouped according to the domains of Meaningful Learning Theory: cognitive, affective and psychomotor [13].

**Fig. 1.**
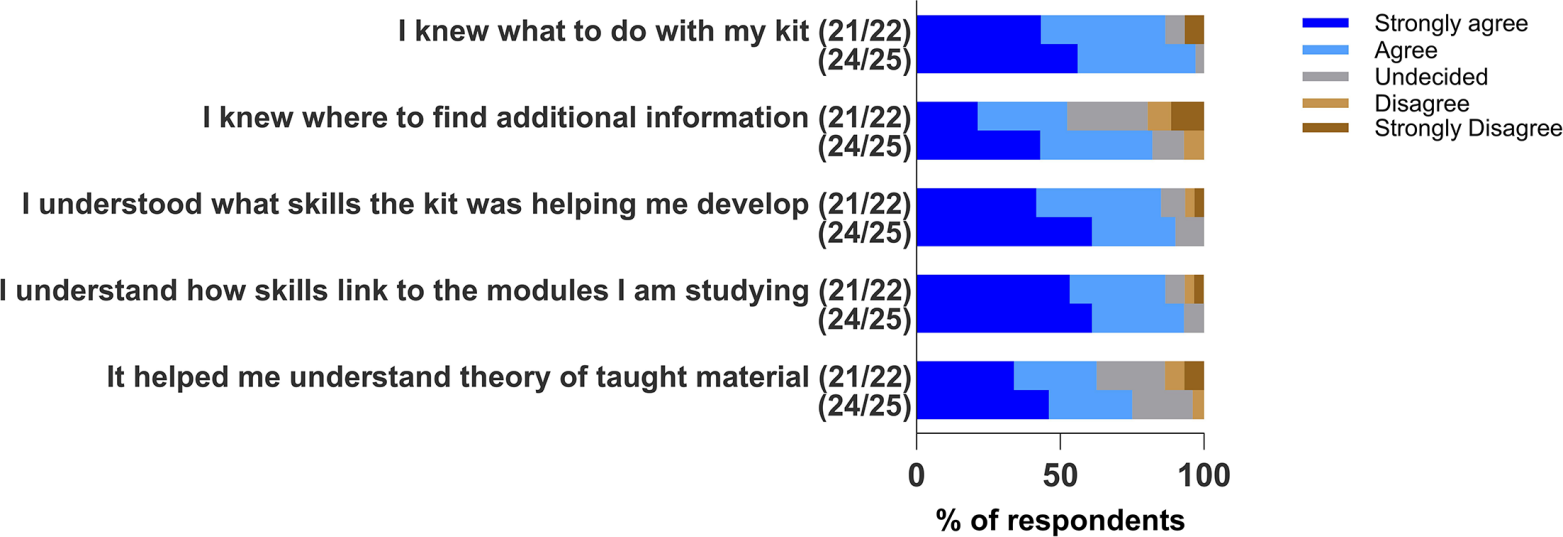
Stacked bar chart showing participants’ responses from two academic cohorts, 2021/22 and 2024/25, to questions on the impact of the Bioskills at home kit on the cognitive domain.

Questions relating to the cognitive domain are represented in Fig. 1. Over 80% of the survey participants in both the 2021/22 and 2024/25 cohorts agreed or strongly agreed to the statements ‘I knew what to do with my kit’, ‘I understood what skills the kit was helping me develop’ and ‘I understand how the skills link to the modules I am studying’. For the statements ‘I knew where to find additional information’ and ‘It helped me understand the theory of taught material’ in the 2021/22 cohort, 52% and 62% of survey participants agreed/strongly agreed, respectively, with higher percentages of 82% and 75% observed for the 2024/25 participants.

**Fig. 2.**
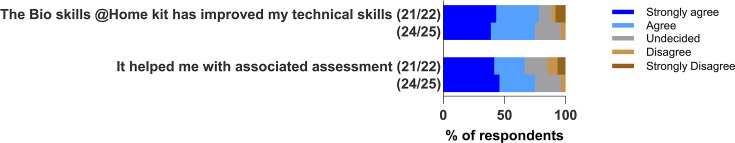
Stacked bar chart showing participants’ responses from two academic cohorts, 2021/22 and 2024/25, to questions on the impact of the Bioskills at home kit on psychomotor skills.

Responses to questions on psychomotor skills are given in Fig. 2. Participants agreed/strongly agreed that the kit has helped improve their technical skills at 78% and 75% in 2021/22 and 2024/25, respectively, and it helped them with the associated compulsory assessment at 66% and 75% for 2021/22 and 2024/25, respectively.

**Fig. 3.**
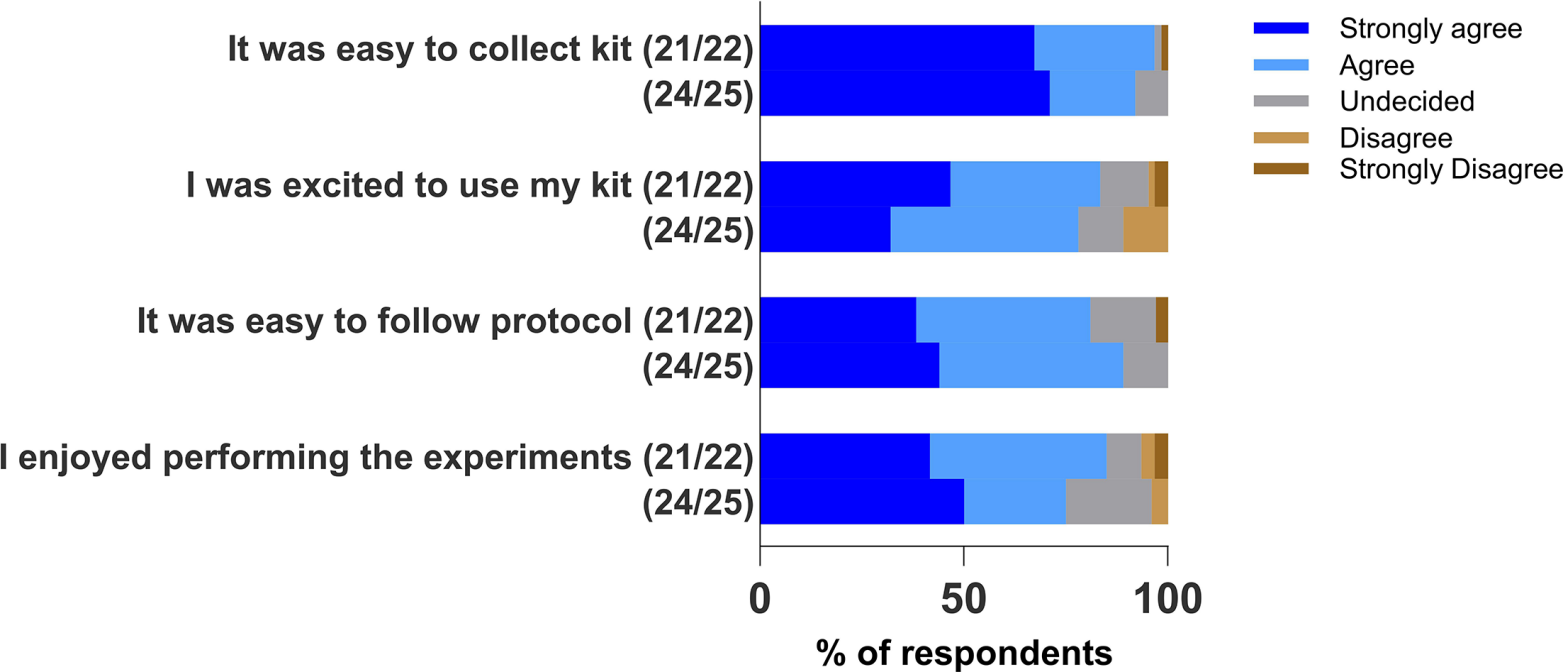
Stacked bar chart showing participants’ responses from two academic cohorts, 2021/22 and 2024/25, to questions on the impact of the Bioskills at home kit on the affective domain (experience, ease and excitement).

For the affective domain (experience, ease and enjoyment) given in Fig. 3, in both 2021/2022 and 2024/25 cohorts, over 92% of the participants agreed/strongly agreed that it was easy to collect the kit, and ≥78% agreed/strongly agreed that they were excited to use the kit; ≥81% agreed/strongly agreed that it was easy to follow the protocol, and over 75% agreed/strongly agreed that they enjoyed performing the experiments.

**Fig. 4.**
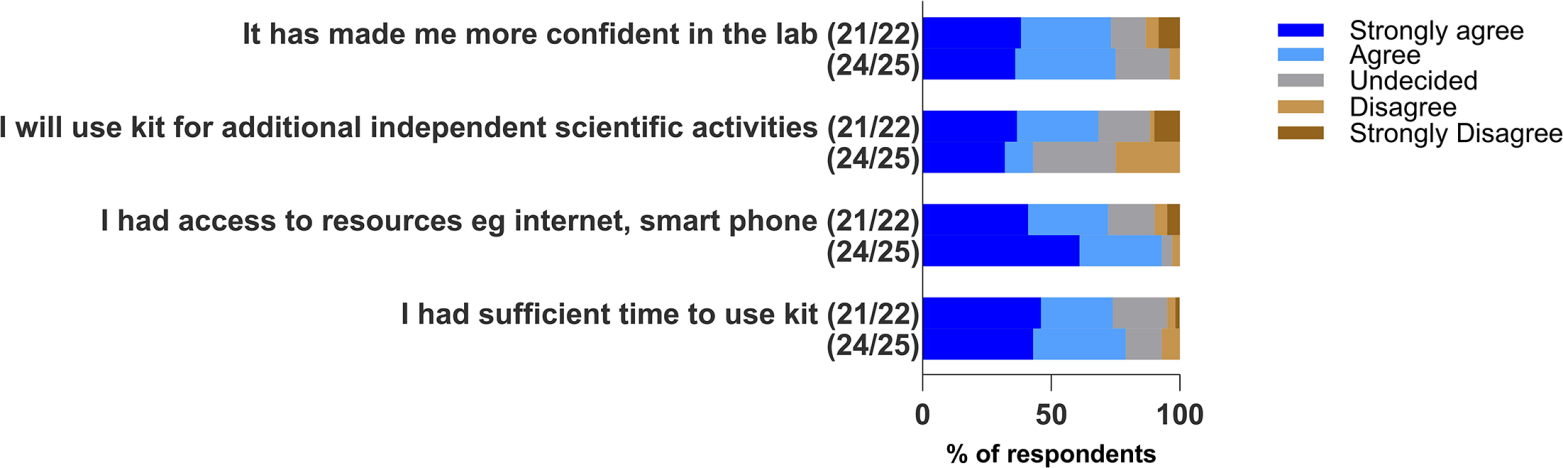
Stacked bar chart showing participants’ responses from two academic cohorts, 2021/22 and 2024/25, to questions on the impact of the Bioskills at home kit on the affective domain (confidence, motivation and challenges).

Considering the affective domain (confidence, motivation and challenges) in Fig. 4, for both cohorts, participants agreed/strongly agreed that they have become more confident in the lab (≥73%). More students agreed/strongly agreed (68%) in 2021/22 that they would use their kits for additional independent scientific activities than in 2024/25 (43%). A Spearman rank order test was performed to identify any relationship between feeling confident and the motivation to use kit independently in 2024/25, and this showed a positive correlation (ρ=0.56, *P*=0.001). There was also a positive statistical correlation between 'I enjoyed performing the experiments' and 'I will use kit for additional independent scientific activities' (ρ=0.44, *P*=0.009). Over 72% agreed/strongly agreed that they had access to key resources such as the internet and smartphones and had sufficient time to use the kit (74%).

**Fig. 5.**
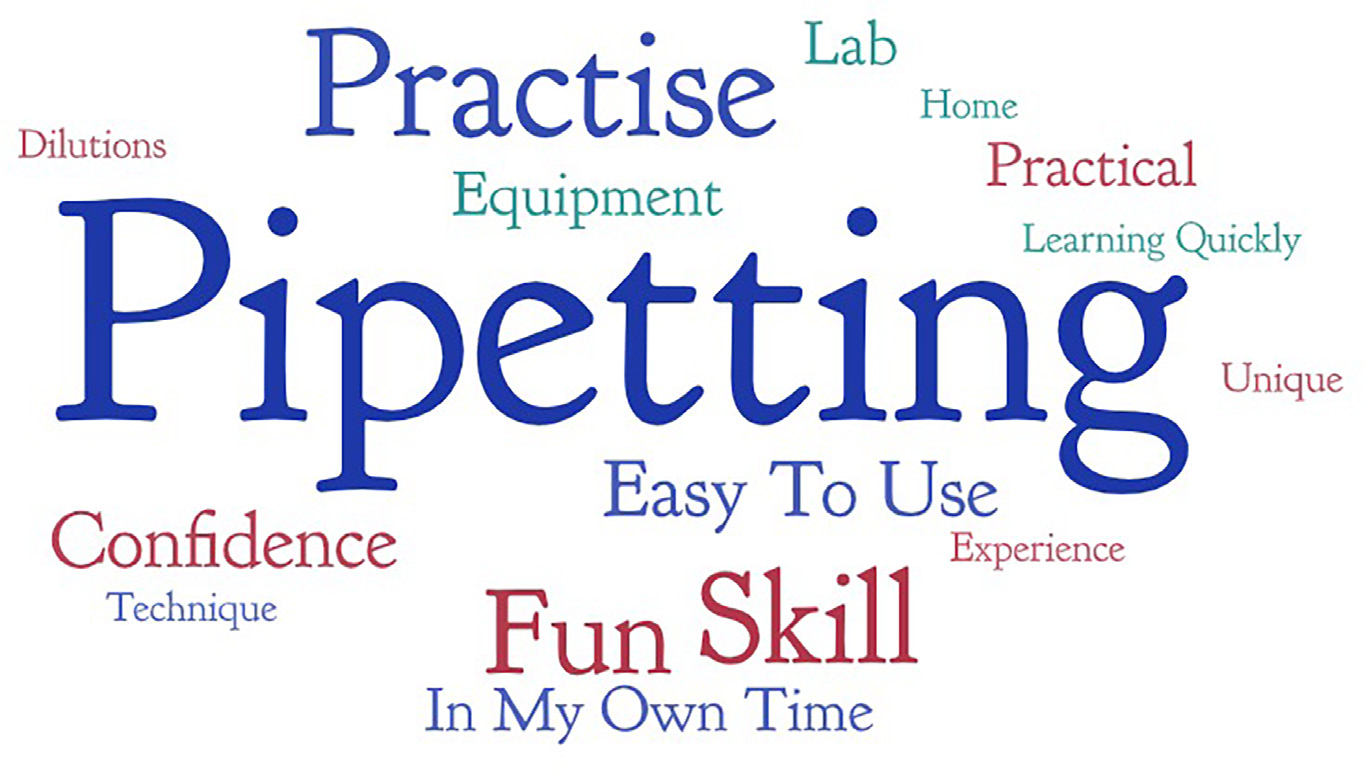
A word cloud visualizing response of students to an open-ended survey question to identify what they enjoyed the most about using the Bioskills at home kit. Image generated using WordArt (www.wordart.com).

An open-ended question in the survey asked participants to identify what they enjoyed the most about using the Bioskills at home kit. The responses were grouped into similar themes and are presented in a word cloud (Fig. 5) which shows the frequency of words and phrases. Occurring most frequently were *pipetting* (30), *practise* (10), *skill* (7), *fun* (6), *easy to use* (3) and *confidence* (3).

### Student attainment

**Fig. 6.**
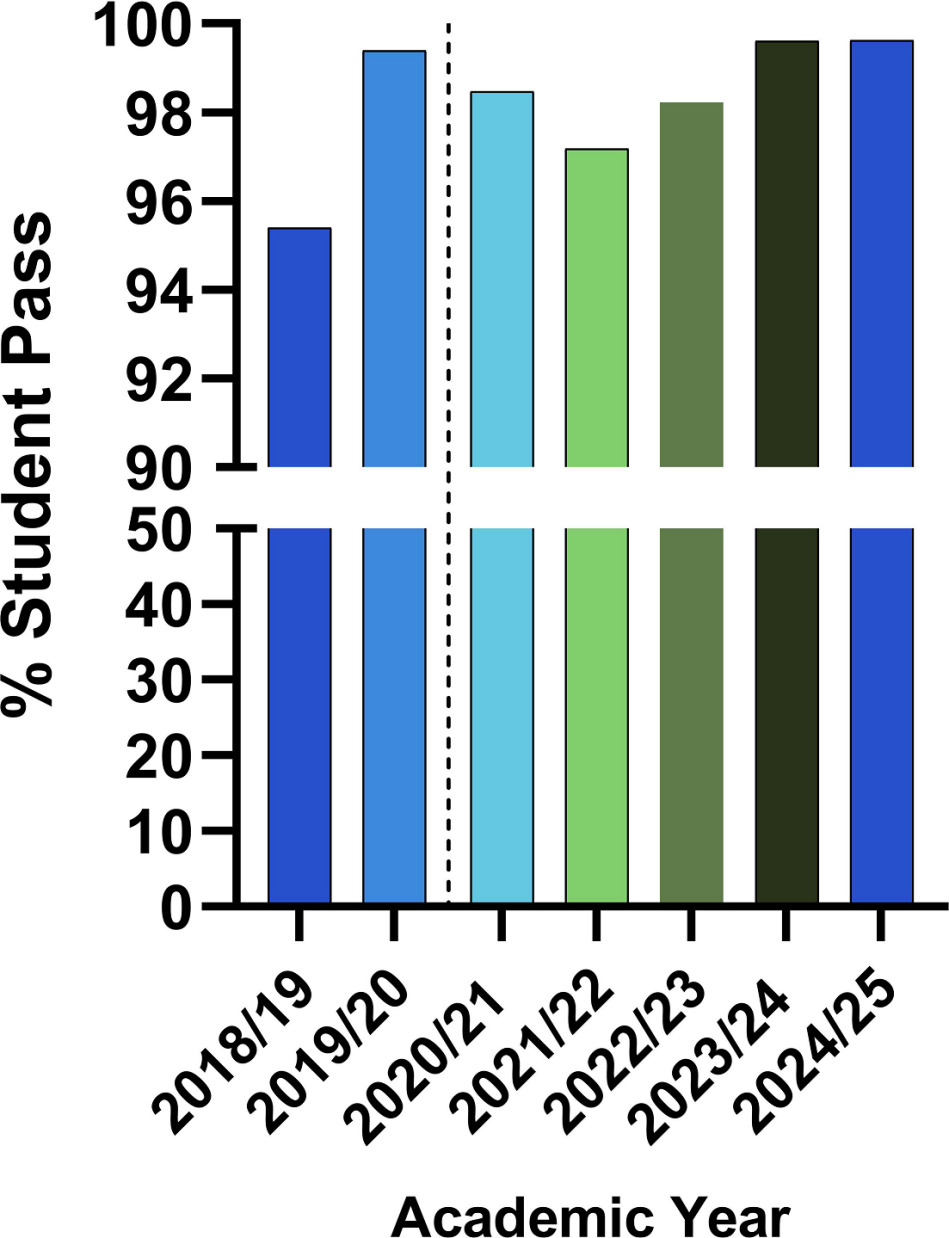
Percentage pass at first sit in the compulsory pipetting assessment in the Practical Techniques module for first-year bioscience students, for seven academic sessions, 2018/19–2024/25. The vertical dotted line separates the bars into pre- and post-intervention/pandemic.

The percentage pass in the compulsory pipetting assessment within the *Practical Techniques* module undertaken by all first-year bioscience students was analysed across 7 years, between 2018/19 and 2024/25. The numbers of students who took this test at first sit were 327, 336, 391, 354, 281, 259 and 273, respectively. Fig. 6 gives the attainment (graded as either pass or fail) of students pre- and post-Bioskills at home kit intervention. The lowest attainment was observed in 2018/19 (95.4%) with an increase of up to 99.6% in 2024/25. Although the baseline pass rates were generally high, the improvement across the years is statistically significant at *P*<0.01 using a Chi-square test.

### Student engagement data

To enable comparison of student engagement with the bioskills activities before and after embedding within the personal tutorial system, the average attendance at the tutorial sessions where the kit activities were delivered, as captured in the NTU online workspace, was measured. Across the 2021/22 and 2024/25 sessions, the average attendance was 66%.

## Discussion

The Bioskills at home kit was originally developed during the COVID pandemic as an intervention to support the development of core laboratory skills and a sense of belonging among first-year bioscience students at NTU at a period when access to the laboratory was limited. Following its initial implementation, some limitations, including a lack of engagement, were encountered. Rayment *et al.* [25] noted a low proportion of students engaging with the Bioskills at home kit online resources based on the Virtual Learning Environment analytics. This challenge led to the embedding of the kit within the personal tutorial system in subsequent years, as a more streamlined iteration. Moving the kit delivery to the tutorials not only ensured students readily received the kit but overcame the initial barrier of engaging with the activities as students could perform the activities for the first time as a group with facilitated tutor support. Previous report noted the engagement with the online guidance and resources to be between 12.7 and 22.9% [25], compared with the engagement data in subsequent years (2021/22 and 2024/25), where 66% of students engaged with the tutorial session designed for the kit delivery. Embedding the delivery in the tutorial session did not only increase student engagement but also created the opportunity to build a healthy sense of community among new university students, and the hands-on pipetting activity served as a fun icebreaker in this tutorial at the start of the first term.

In the first year which was during the pandemic, the students were not attending campus and did not have the opportunity to meet their colleagues, contributing to the sense of isolation and reduced engagement. A crucial aspect to the process of learning is a social environment which is that sense of community that allows interaction, exchange and construction of knowledge highlighted by the theory of social constructivism [32]. By creating space, time and structured support for tutees to interact as a community, engagement with the kit could be enhanced. The delivery of tutorials was standardized across all tutor groups by tutor training and provision of a common guidance sheets. This ensured parity in student experience at personal tutorials. The Bioskills at home kits contained instructions on how to carry out associated experiments while at home, and the expectation was that students through regular home practice of these skills would familiarize themselves with the protocols and develop both competence and confidence when they get into the laboratory.

Deep and authentic learning takes place when students are able to make meaning of the knowledge they receive, and there has to be the integrative reconciliation of prior knowledge within new learning for lasting cognitive connections to be achieved. Novak [13] posits that meaningful learning takes place when new knowledge is deliberately linked by the learner to existing concepts. This is particularly the case in the laboratory as students seek to understand theoretical concepts by actively engaging in practical experiments. To achieve this, there is an interplay of the cognitive, affective and psychomotor domains of learning.

### Assessing impact of Bioskills at home kit on cognitive domain

Survey questions to determine the impact of the Bioskills at home kit on the knowledge and intellectual skills of the participants show that students had a very good (≥80% agree/strongly agree) understanding of what to do with their kit based on resources made available to them. It was clear to them what skills the kit was helping them develop, and they were able to make the connection between the kit activities and their modules. The laboratory classes offer a range of benefits to students [33] and represent an environment for students to experience and create knowledge as they participate in hands-on learning. Particularly for first-year bioscience students, abstract theoretical concepts which have been taught within modules come alive as they have concrete experience and engage in both active experimentation and reflective observation in the lab [9].

As the delivery of the kit was transitioned to being embedded within the tutorials after the 2020/21 session, it was expected that students would feel better supported by personal tutors and be more easily signposted to any additional resources they needed for their learning. For the 2020/21 cohort, all participants of the focus group noted a lack of confidence about where to find support for these activities; consequently, suggestions such as smaller subject-specific discussion boards to encourage engagement were made (Table 2). In the subsequent cohorts, we see an improvement of this to 52% and 82% (2021/22 and 2024/25, respectively) of the students who understood where to find additional information and 62% and 75% (2021/22 and 2024/25, respectively) of students who agreed that the kit helped them understand the theory of the taught material. This supports the role of the tutorial system in the effective delivery of the Bioskills at home kit to enhance student knowledge and bridge the gap between theoretical concepts and practice. Compared with 2024/25, these percentages are observed to be lower in the 2021/22 session. Being the cohort just coming out of the COVID pandemic, the 2021/22 cohort appears to have been most affected as also noticed in the student attainment outcome in the compulsory pipetting assessment (Fig. 6). Additionally, the 2024/25 cohort was fully back on campus, unlike the 2021/22 cohort, and would have had considerably more support from tutors.

### Assessing impact of Bioskills at home kit on affective domain

There is a dynamic relationship between emotional (affective) and cognitive learning processes. Successful cognitive learning can often contribute to improved emotional responses and vice versa. Consequently, participants’ experiences such as ease, enjoyment, excitement, confidence, motivation and challenges were explored in this study. In the 2021/22 and 2024/25 cohorts, the majority of the survey respondents (at least 92% and 81%, respectively) agreed that it was easy to collect the kit and follow the protocol. They enjoyed performing the experiments (≥75%) and were excited to use the kit (≥78%). Although the COVID-19 situation in 2020/21 made collecting the kits challenging and these had to be posted to students wherever they were around the world, participants agreed that they enjoyed performing the experiments and were excited to use the kit as demonstrated in the quote below:

‘*I think everyone was actually like interested and wanted to try it because it was like a cool new iPhone.*’

Emotions such as enjoyment have been shown to have positive influences on learning as they evoke strong memories, facilitate deep learning and boost confidence [34]. The focus group data showed that the 2020/21 cohort was particularly engaged in the microscopy activity and thought the microscopy competition and celebratory event were fun. They also mentioned enjoying the pipetting activity. Additionally, from Fig. 5, the most frequently occurring words from the open-ended survey questions describing aspects of enjoyment were pipetting, practise and skill. The pipetting activity was the most enjoyed among the 2021/22 and 2024/25 cohorts. Furthermore, a moderately strong positive correlation between kit enjoyment and motivation to use kit again is demonstrated at a significant level of *P*=0.009.

The students also appreciated the opportunity to engage in sufficiently challenging activities, as it made them use an unfamiliar object in a comfortable environment.

The bioskills kit was able to improve participants’ confidence in the laboratory (75% and 73% in 2021/2022 and 2024/25, respectively), which was a desired goal when the bioskills kit was being developed. Confidence was also one of the commonly occurring words when the students were asked what they enjoyed most about the Bioskills at home kit (Fig. 5). Self-efficacy, which describes students’ confidence in their abilities to perform lab tasks, is key for improving performance and has been shown to strongly correlate to successful outcomes [35,36]. Aside from contributing to successful outcomes, confidence can have a significant impact on their possible career directions. When students are able to view themselves as competent in specific lab skills, it can inspire enduring interest in the subject, which can influence their choice of future careers. This strengthens the individual’s science identity as described by the social cognitive career theory [37,38].

Respondents (68%) agreed that they would use their kits for additional independent activities in 2021/22, while 43% agreed in 2024/25. Although there was a positive correlation between feeling confident and motivation to carry out independent activities with the kit in 2024/25 (*P*=0.001), some students mentioned that they did not feel the need to do extra with the kit at home as they were already having a significant number of hours to practise skills in the laboratory within various modules. They also noted that they tried to exercise caution by limiting the use of the pipettes, recognizing that these were to be returned at the end of the academic year. This may have contributed to the lack of enthusiasm in exploring additional activities with the kit. In contrast, the 2021/22 cohort, which still experienced some limitations with access to all laboratory sessions, was more motivated to use their kits for additional independent activities.

From the survey and focus group data, barriers to using the kit include limited time, balancing workload demands as new university students, clashing priorities, access to resources such as internet and smartphone, technical challenges with the kit such as ‘*the yeast did not grow*’ or just a lack of interest. Particularly with the 2020/21 cohort, there was the fear of getting it wrong at the discussion boards and being judged by course mates they had never met, which contributed to the lack of engagement in the online platform we provided. Transitioning the kit delivery to the personal tutorial system was able to address some of these issues in later years.

### Assessing impact of Bioskills at home kit on psychomotor domain

Within the biosciences lab, technical skills such as accurate pipetting, microscopy and growth curve are foundational to the successful student journey throughout the university as a number of laboratory experiments require proficiency in these skills. These practical skills enable students to incorporate their personal experience to enhance cognitive engagement and deeper learning according to Novak’s theory of meaningful learning [13]. Typically, at NTU, first-year students would have between 80 - 90 h of lab sessions; however, during the COVID-19 pandemic, this was significantly reduced to between 0 - 15 h which led to the initial development of the bioskills kit aimed at supporting students to develop these core skills. The use of the bioskills kit was continued post-pandemic due to its perceived benefits in enhancing student lab proficiency. Consequently, one of the goals of this study was to ascertain if the kit had supported the development of these skills.

Data from the focus group, survey and student attainment suggest that the kit promoted the development of core lab skills among participants. Exploring the benefits of the Bioskills at home kit, participants from the first student cohort (2020/21) affirmed the advantage of the kit in developing lab skills which they otherwise would not have had the opportunity to practise. This benefit was directly relevant to the associated *Practical Techniques* assessment and supported their success in this test. This was also the case from the survey for the 2021/2022 and 2024/25 cohorts as 78% and 75%, respectively, of participants agreed that the kit has helped improve their technical skills. To further support this, the 7-year data on student attainment on the assessment show the percentage of students who passed the test at first sit to be 95.4% in 2018/19 (pre-intervention) and up to 99.6% in the 2024/25 session (post-intervention). The baseline attainment to compare the outcome of the kit intervention with would be that of 2018/19 as there was a one-off intervention in place (separate from this work) during 2019/20 [3], involving pre-lab sessions which could not be rolled over to the 2020/21 during the pandemic, as students could not attend the lab. What is worth noting here is that during the pandemic where students had no lab access, they were able to maintain a level of competency above the 2018/19 level.

From the results obtained in this study, there is evidence to show that the Bioskills at home kit was able to support the students’ learning across the cognitive, affective and psychomotor domains of learning. Embedding the delivery of the kit within the tutorial system helped in overcoming the challenge of lack of engagement encountered during the pandemic when the first iteration of the kit was launched, and dedicated tutor guidance also meant students could be better supported with the use of the kit.

The issue of sustainability raised by the first cohort was addressed by streamlining the kit content and successfully establishing a return back policy. This system has been successful so far. The Bioskills at home kit has been effectively implemented at NTU for 5 years, and further developments include expanding this initiative into portable class packs which can be taken into secondary schools and the wider community for science outreach activities to inspire the next generation of bioscientists. There is also potential to build skills capacity in under-resourced communities and to extend the Bioskills at home kit through international collaborations.

### Limitation of study

The comparatively small number of participants for the focus group and survey is acknowledged, and more robust feedback would have been obtained with higher response rates. Focus group and survey participation were entirely voluntary, and students chose to engage or not with the knowledge that it has no impact on their studies or assessment grades. The relatively close response rate for the survey, 17% (2021/22) and 10% (2024/25), however, meant that the data collected could be comparable. In addition, enjoyment and engagement with the kit could not be directly correlated with individual student attainment as survey was anonymous.

### Conclusion

The Bioskills at home kit represents an innovative response to the challenges posed by restricted laboratory access during the COVID-19 pandemic, offering valuable learning opportunities for first-year bioscience students. Situated in the experiential and meaningful learning theories, the kit supported the development of core laboratory skills in a familiar environment, fostering confidence, competence and a sense of belonging. Embedding the kit within the personal tutorial system provided a more structured and efficient delivery system that significantly improved student engagement which had a favourable impact on student experience and attainment. Survey and focus group data across multiple cohorts demonstrate positive effects on the cognitive, affective and psychomotor domains. Qualitative and quantitative data show that students experienced improved comprehension of theoretical concepts, developed technical skills and found the kit easy and enjoyable to use. Embracing the evolving dynamism of higher education requires adaptability in our provision of learning options for our students, beyond on-campus activities. The successful implementation of the Bioskills at home kit demonstrates a scalable model for flexible laboratory teaching.

## Supplementary material

10.1099/acmi.0.001157.v3 Supplementary Material 1.

10.1099/acmi.0.001157.v3 Supplementary Material 2.

## Abbreviations

NTU: Nottingham Trent University
