# Supplementary material for: Evaluating the impact of a laboratory teaching innovation: the case of the Bioskills at home kit
Source: Access Microbiol. 2026 May 21;8(5):001157.v3. doi: 10.1099/acmi.0.001157.v3 (PMC13193619; doi:10.1099/acmi.0.001157.v3)
Supplement: Supplementary Material 1. [file acmi-8-01157-s001.pdf]

FOCUS GROUP

Topic list

Research goals of the focus group:

- To explore students' differing experiences of engaging with the NTU Bioskills at home kit.
- To better understand possible barriers of engagement with the kit so that these can be removed in future versions
- To investigate how the kit and its delivery could be improved in future, including:
    - Ease of use, enjoyability, motivation, perceived benefit, support available

Focus group part 1: Ice breaker (Approximately 5 minutes)

Purpose: Welcome students to the focus group, introductions, explain aims of the focus group, housekeeping rules, explain that the Teams meeting is recorded and recap on consent and right to withdrawal agreement.

Focus group part 2: (Approximately 15 minutes)

This section focuses on exploring student's feelings around initially engaging with the kit.

The researcher will ask the students what their initial impressions were on collecting their kits, this can include prompts such as:

- How easy was it to collect their kits, could this be improved?
- How did the students feel about receiving their kit?
- What did students think about what was included in the kit?
- Was it clear what the purpose of the kits was when they were collected?
- Did the students feel motivated to use the kit – what could have motivated them more?
- Was there anything in the kit the students were particularly impressed with?
- Was there anything students felt was missing from the kit?

Focus group part 3: (Approximately 15 minutes)

This section focuses on students' experiences of particular tasks they were asked to perform using the kit

Broad questions include the following, researcher can probe for more detail

- Did the students particularly enjoy any of the individual tasks set – if so which one and why?
- Was there any particular task the students didn't enjoy (most difficult) – which one and why?
- How did students find the pipetting exercise?
- How did students find the growth curve experiment?
- How did students find the haemocytometer exercise?
- What were the students' impressions of the microscopy competition? Did they enter? If not, why?

Focus group part 4: (approximately 15 minutes)

This section focuses more specifically on how students feel the kit could be improved and supported

Researcher will explain that our hope was to get as many students to engage with their kits as possible and this would provide students with the opportunity to build contacts and a sense of community with their peers through discussion. This is something we felt would be important with lockdowns affecting their opportunities to interact.

We had envisaged that using the kit would be fully flexible process – with students engaging with the kit at times which suited them. We want to learn what worked and didn't work them – this might include more structure to using the kit. Prompts / questions to ask could include:

- If students were designing their ideal kit, what would they include?
- Did the students feel it supported their degree? What other experiments would they have liked?
- Could students see the link between the proposed experiments in the kit and their taught material.
- Is there anything they would like to have more practice of? This doesn't have to be "physical" experiments, e.g. it could be data analysis, calculations, use of excel etc,
- What would have motivated them to use their kit more?
- How could support for using the kits be improved? Videos, timetabled sessions, more detailed worksheets, student work groups where they perform experiments together

Focus group part 5: (approximately 5 minutes)

This section will allow students to discuss anything not covered directly by the topic list

The researcher will ask the students if they have any other comments or suggestions they would like to discuss or provide. This provides students with a chance to provide possibly important information not considered by the researchers.

Wrap-up: (Approximately 5 minutes)

Thank students for participation, Recap positive aspects of the session, remind students that this type of reflection can help them going forward with their studies.

Remind students about limitations of withdrawing from study

Direct students to student support services should any of the issues discussed have affected them in any way.

Focus Group: Participant Information Sheet

Thank you for your willingness to participate in this focus group. Before progressing, please make sure that you are familiar with the conditions of participation:

Project purpose

The purpose of the focus group is to explore students' experiences of engaging with the Bio Skills @Home kit and how we can improve it for future years. This study has been approved by the School Of Science & Technology Non-invasive ethics committee at NTU.

Participants will be provided with a £10 Amazon voucher **on completion of the focus group** in recognition of their time they have given up to be able to`participate in the study. Participation in this study will not affect your study or attainment achieved as part of your course at NTU.

Right to withdraw

Your participation in this focus group is entirely voluntary and will have no impact on your studies. You have the right to withdraw from the study up until the focus group takes places and at any point during the focus group. However, you will not be able to withdraw any data from the completed group discussion, as this will be an integral part of the group narrative and will not be distinguishable/separable from data provided by others focus group members.

You can withdraw by using any of the contacts below:

bunmi.omorotionmwan@ntu.ac.uk

david.negus@ntu.ac.uk

Data collection, use and storage

Data will be collected by audio **recording of the focus group**. The data gathered will be used to improve the Bioskills at home kit and may be used to produce future pedagogic publications. Data (voice recordings and transcription of the meeting) will be stored for one year and managed using NTU DataStore in line with NTU's Research Data Management Policy.

Anonymity

Every reasonable step will be taken to maintain your anonymity and confidentiality. Any quotes or statements provided by you will be anonymised in any future publications. Participants should not discuss other participant's contributions outside of the focus group.

Participants will be allocated a unique participant identifier.

YOUR PARTICIPANT IDENTIFIER IS ………………………………………………….. (not required for this focus group as they are made to understand they can't opt out after the session is over)

Participant Consent Form

Dear Research Participant,

If you are happy to take part in this focus group, please read the following statements and tick the boxes where appropriate:

I have read the participant information sheet and had an opportunity to ask questions about the research and received satisfactory answers to any questions.	☐
I have had sufficient information to decide whether or not I wish to take part in the study.	☐
I understand that my grades on my course will not be affected by whether or not I choose to participate in this study	☐
I understand that the focus group will be recorded.	☐
I understand that I am free to withdraw from the focus group at any point up to the start of the meeting or during the meeting but that I will not be able to withdraw any data from the completed group discussion.	☐
I agree to take part in the study.	☐
I understand that data, which will be made anonymous, obtained during this study may be included in material published from this research.	☐
I will not discuss the contributions made by other focus group members outside of the focus group.	☐
I understand that I will be eligible for a £10 Amazon voucher on completion of the focus group	☐

Initials
Name
Signature
Date

Bioskills at home Survey

Participant Information

Thank you for your willingness to complete the questionnaire. Before progressing, please make sure that you are familiar with the conditions of participation:
Project purpose: The purpose of the questionnaire is to explore students' experiences of engaging with the Bioskills at home kit and how we can improve it for future years. Right to withdraw Your participation in this survey is entirely voluntary and will have no impact on your studies. You have the right to withdraw from the study until (include date). You can withdraw by providing your unique identifier through email or written letter to the following correspondence: bunmi.omorotionmwan@ntu.ac.uk (Bunmi Omorotionmwan, Nottingham Trent University, Clifton Campus, College Drive, NG11 8NS). The unique identifier will also be used to identify winners of the £10 Amazon vouchers. Anonymity- Every reasonable step will be taken to maintain your anonymity and confidentiality. You will be asked to provide a unique identifier of your choice, this should be a combination of two memorable words. This is so that should you chose to withdraw, your response can be identified whilst protecting your anonymity. Data collection, use and storage Data will be collected using NTU's online survey platform (JISC). The data gathered will be used to produce a pedagogic publication exploring and sharing the impact of the Bioskills at home kit. It will also be used to improve how we deliver the kit in future years. Data (electronic questionnaire results) will be stored for one year and managed using NTU DataStore in line with NTU's Research Data Management Policy. By selecting continue, you are indicating that you fully understand the above information and agree to participate in this study on the basis of the above information.

Participant Consent

1. Dear Research Participant, If you are happy to take part in this project, please read the following statements and tick the boxes to agree:

- I have read the participant information sheet (previous page) and had an opportunity to ask questions about the research and received satisfactory answers to any questions.
- I have had sufficient information to decide whether or not I wish to take part in the study.
- I understand that I am free to withdraw from the research project by (include day/time)
- I agree to take part in the study.
- I understand that data, which will be made anonymous, obtained during this study may be included in material published from this research.

Unique identifier

2. To maintain your anonymity and confidentiality, please provide a unique identifier. This should be two memorable words. This is so that should you chose to withdraw, your response can be identified whilst protecting your anonymity

Survey

3. Please confirm which qualifications you hold
   A-Levels/BTEC/Combination of A-levels and BTEC/Other

4. I used my Bioskills at home kit after collecting it
   Yes/No

5. If you selected No, please tell us why you didn't use the kit:

6. Please rate the following questions / statements about the Bioskills at home Kit using the following scale:
   Strongly agree/Agree/Undecided/Disagree/Strongly Disagree

   6.1 I found it easy to collect my Bioskills at home kit

   6.2 I knew what to do with my Bioskills at home kit

   6.3 I knew where to find out additional information on what I could do with my Bioskills at home kit

   6.4 I had enough time to use my Bioskills at home kit

   6.5 I had easy access to additional resources required to use my Bioskills at home kit (e.g. a smartphone, computer, internet connection)

   6.6 I was excited to use my Bioskills at home kit

   6.7 I found it easy to follow the protocols for the experiments

   6.8 I enjoyed performing the associated experiments eg the pipetting olympics during the tutorials

   6.9 I will use my Bioskills at home kit for additional independent scientific activities

   6.10 I understand what skills the Bioskills at home kit is helping me develop

   6.11 I understand how those skills link to the modules I am studying

   6.12 The Bioskills at home kit has improved my technical skills eg pipetting

   6.13 Using the Bioskills at home kit has made me more confident at working independently in the lab

   6.14 Performing the experiments helped me understand the theory of the taught material

   6.15 Using the kit helped me with an associated assessment (e.g. practical techniques)

Improving the Bioskills at home Kit

7. What did you enjoy most about using the Bio Skills Kit?
8. What was the biggest barrier to using the Bio skills kit?
9. What would be beneficial in supporting you using the Bioskills at home kit?
