# Supplementary material for: Evaluating the impact of a laboratory teaching innovation: the case of the Bioskills at home kit
Source: Access Microbiol. 2026 May 21;8(5):001157.v3. doi: 10.1099/acmi.0.001157.v3 (PMC13193619; doi:10.1099/acmi.0.001157.v3)

Bioskills @Home Kit - Home Exercises and Protocol

Jody Winter, Bunmi Omorotionmwan, Sarah Rayment, Karin Garrie, Ishwinder Kaur, Gareth McVicker

Department of Biosciences, Nottingham Trent University, Nottingham, UK

This document contains the home exercises and protocol associated with the Bioskills @Home kit

Table of Contents

Bioskills @Home Kit Content 2

Initial Version 2

Streamlined Version 2

Exercises and Protocols 4

Microbial growth curve experiments 4

Micropipette home exercises 8

How to set up microscope at home 14

Haemocytometer as a scale 18

Listed below are the contents of the Bioskills @Home kit- both the initial version and subsequent streamlined iteration.

Bioskills @Home Kit Content

Initial Version

Supplied to each student in an NTU branded canvas bag:

- Welcome leaflet
- Digital microscope
- Micropipette 2-20 µl
- Micropipette 20-200 µl
- Micropipette 200-1000 µl
- 1 x permanent marker pen
- Safety glasses
- 4 glass microscope slides and 4 cover slips per student (perhaps wrapped in a piece of tissue paper and taped inside a petri dish? – label with warning that contains glass).
- 1 additional empty petri dish
- 10 pasteur pipettes
- 8 x 50 ml falcon tubes
- 2 x C-chip disposable haemocytometer slides
- 22 x 1.5 ml Eppendorf tubes
- 6 x pairs of gloves (medium)
- Dried yeast (~0.5 g in an Eppendorf tube)
- 40 x yellow tips
- 40 x blue tips
- Filter paper (3 sheets, each approx. 15 cm x 15 cm)
- Yellow top tube labelled "Dye Solution Concentrate" containing 20 ml blackcurrant squash concentrate
- Ink (either 2-3 black fountain pen ink cartridges per student, or 5 ml black ink supplied in a tube)
- Isopropyl alcohol 10 ml per student, supplied in a labelled tube
- 1 x tweezers per student (scientific tweezers/forceps are not required, so these could be bulk purchased cheaply from Amazon)
- 3 x small disposable plastic weighing boats. Ideally square and flat bottomed – to be used as staining vessels for onion skin cell staining with ink. Something like VWR 611-0093 would be ideal.

Streamlined Version

- Micropipette 20-200 µl
- 1 additional empty petri dish
- 8 x 50 ml falcon tubes
- 22 x 1.5 ml Eppendorf tubes
- Dried yeast (~0.5 g in an Eppendorf tube)
- 40 x yellow tips
- Filter paper (3 sheets, each approx. 15 cm x 15 cm)
- Yellow top tube labelled "Dye Solution Concentrate" containing 20 ml blackcurrant squash concentrate

Bio Skills @Home learning pack contents guide

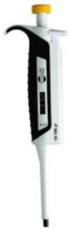
Micro-pipettor (pipette)

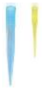
Pipette tips

Pasteur pipette

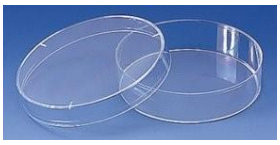
Petri dish & lid

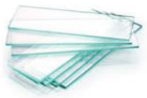
Glass microscope slides

Glass cover slips

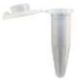
1.5 ml Eppendorf or microfuge tube

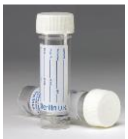
25 ml universal tube (may have yellow cap)

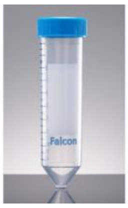

50 ml falcon tube

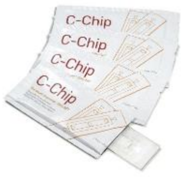

Haemocytometer slides

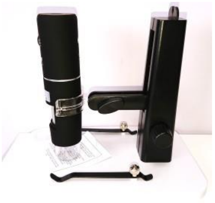
Digital microscope

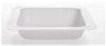
Weigh boat (for use in tissue staining)

Pictorial representation of some contents of the bioskills @home kit

Exercises and Protocols

Microbial growth curve experiments

What is a growth curve?

A growth curve is simply a graph of the number of microbial cells present in a nutritious growth medium over time. This number can be measured directly (such as by plating out samples to see how many microbes grow) or indirectly (such as through the observation of turbidity, i.e. how cloudy the sample is). This experiment will show you how to produce a growth curve with the materials in your kitchen and the Bio Skills @Home pack.

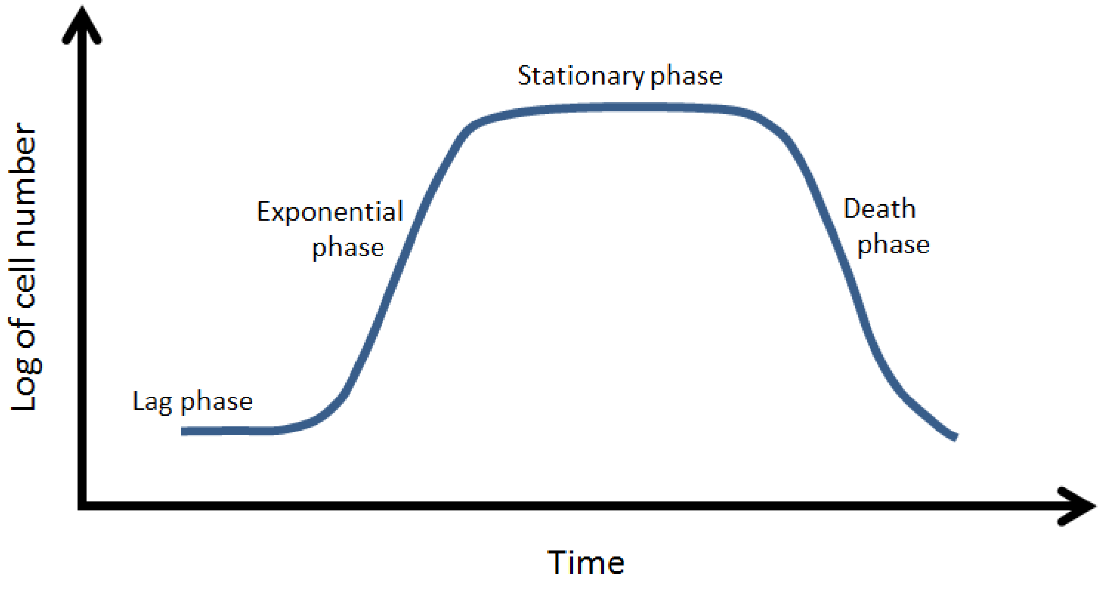

Preparing to complete this activity

You will need the following from your pack:

- Dried *Saccharomyces cerevisiae* (baker's yeast). This is a harmless, eukaryotic microorganism that humans have used in baking, brewing and medicine for centuries.
- 6x 50 ml centrifuge tubes ("Falcon tubes").
- 1x 1.5 ml microcentrifuge tube ("Eppendorf tube").
- 1x Pasteur pipette.
- A permanent marker for writing on your tubes.
- The quantification system from your pack (a printed sheet showing a fading column of numbers from 10 to 0). If needed, you can print a scale, pdf included in resource pack – Growth Curve Scale

You will need to provide the following from your own kitchen:

- Room-temperature tap water.
- A kettle or saucepan for boiling water (alternatively, you can use very hot tap water for this part instead).

- Sugar.
- A soluble food/drink that is rich in nutrients (e.g. Marmite, clear apple juice).
- A mug or jug.
- A teaspoon.

WARNINGS:

- Use extreme caution when dealing with scalding hot water, electricity or flames – it's easy to burn yourself or cause an accident if you're not paying attention.
- Never consume any part of this experiment – whilst your yeast is harmless, you never know what contaminants are growing in your sample that could very cause severe illness!
- Do not perform this experiment in areas where food is prepared – there is a small risk of making yourself or others sick.
- Do not open your tubes once you have inoculated them – again, you may be growing some dangerous contaminants that could make you very ill. When you have completed your experiment, dispose of your sample tubes as regular kitchen waste without opening them.

Growth curve protocol

The following protocol will allow you to observe the effect of table sugar on the growth rate and growth yield of your yeast. It will take around 10-12 hours, during which time you will need to check your samples at regular intervals (e.g. once per hour). **Read through the entire protocol before you start, so that you know what to expect.**

You should feel free to add or substitute ingredients if you wish to test their effects. Steps 5 and 6 of Part One are the point at which you will need to consider ingredient additions/substitutions – just remember to include the proper experimental controls!

Some good examples experiments might be:

- Try using artificial sweetener instead of sugar to see if it supports microbial growth.
- Try adding garlic extract, diluted toothpaste or mouthwash to see if they slow microbial growth.
- Try incubating your samples at different temperatures whilst keeping your medium identical.

Note the point at which you add sugar (Part One, Step 5). If you want to replace sugar with something else, you will need to pour off some spare medium before that point. Plan your experiment carefully!

Be inventive and have fun!

Part One: Preparing your growth medium

1. Boil approximately half a litre of tap water (or alternatively use very hot tap water for the next step).

2. Measure out approximately 300 ml (about one mug's worth) of boiling water into your mug or jug.

3. Mix your nutritious food (e.g. one quarter teaspoon of Marmite) into the hot water. Your growth medium should be as clear as possible (i.e. with no obvious particles suspended in the mixture; not cloudy) but does not need to be colourless. If using Marmite, your growth medium should be a clear, golden-brown colour.

4. Whilst your growth medium is still hot, very carefully pour 30 ml into each of two sterile centrifuge tubes. Try to work as cleanly as possible so you don't contaminate the medium. Label both tubes as "growth medium only". Label one tube as "yeast" and the other as "no yeast".

5. Add one heaped teaspoon of sugar to the remaining medium in the mug and stir it until it is fully dissolved. Again, whilst the medium remains hot, very carefully pour 30 ml into each of two sterile centrifuge tubes, being as clean as possible. Label both tubes as "growth medium plus sugar". Label one tube as "yeast" and the other as "no yeast".

You should now have four tubes, labelled as follows:
Growth medium only – yeast
Growth medium only – no yeast
Growth medium plus sugar – yeast
Growth medium plus sugar – no yeast

6. You have enough growth medium and tubes remaining for two more samples (a maximum of six tubes in total), if you wish to add your own ideas to the experiment.

7. Allow all of your tubes to cool down to room temperature before you inoculate them in Part Two, or you might accidentally kill your yeast!

Part Two: Inoculating your medium with the yeast

1. Add approximately 0.5 ml room temperature tap water into a sterile 1.5 ml microcentrifuge tube.

2. Add a few grains of dried yeast to the water, close the lid and mix thoroughly by shaking. Keep adding yeast in small amounts until the colour of the solution is like watery milk. Try not to allow any lumps to remain after mixing.

3. Using a Pasteur pipette, add one small drop of the homogenous yeast mixture to each tube that you've labelled "yeast". Try to be consistent with the amount you add to each tube.

4. Do not add any yeast to the tubes marked "no yeast", as these are your uninoculated controls. You will need them to see if your growth medium was contaminated at any point.

5. Loosen the lids on all your tubes a little. They should not be open fully but should be loose enough for gas to escape, to avoid any explosions as the yeast ferment the medium!

Part Three: Perform the growth curve

1. As soon as the yeast has been added to your samples, hold up each tube approximately 2 cm in front of your quantification scale and record the lowest (faintest) visible number that you can read through each individual tube. You can take a photo if you wish! If your samples are relatively clear at this point, you should easily be able to see lowest numbers (0, 1 or 2). We call this initial reading "t = 0" or "time zero"; the starting point of the experiment.

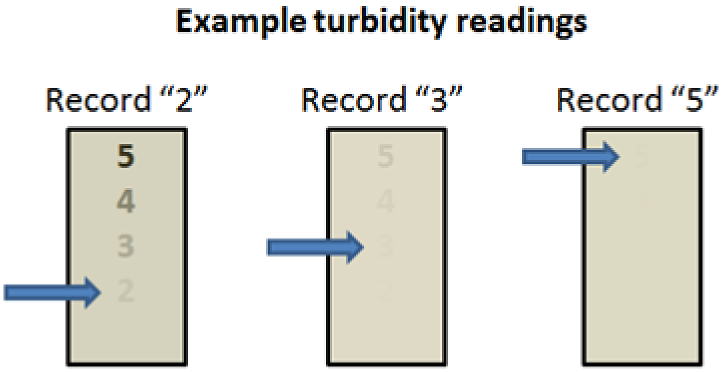

(These example images will depend upon your screen's brightness setting!)

2. Incubate your tubes at room temperature or in a slightly warm place (such as near a radiator). Don't put them into direct contact with a heat source.

3. Perform your measurement again after precisely one hour has passed (t = 1), then again after each subsequent hour up until approximately t = 12. Each time you take a reading, try to keep the tubes the same distance from the quantification scale, as distance will affect your reading. If your yeast is growing (and so the medium is becoming turbid/cloudy), the lowest visible number should increase over time.

4. When you have finished your experiment, try plotting your readings onto a graph using Excel or a similar software package. Plot time in hours on the x-axis and quantification number on the y-axis, with each tube as a separate line/curve. You don't need to use a semi-logarithmic plot for this experiment, as your readings are approximate and aren't directly proportional to the number of cells in the sample (unlike spectrophotometer readings).

- Did your "no yeast" controls show any growth? If so, why do you think that is?
- Did adding sugar improve or reduce the yeast's growth rate?
- Did you try any other experiments? If so, what happened?

We would love to see how you get on with this activity. Please post your results on the discussion forum

Remember: **Do not** fully open your tubes once your growth curve has started. When you've finished, dispose of your tubes along with your usual kitchen waste. Leave the lids **slightly loose** so that gas can escape the tubes and prevent any explosions.

Micropipette home exercises

These specialised pipettes are used to dispense microliter volumes (as suggested by the name “micro” pipettes). Mastering the technique and correct use of micropipettes is essential in all aspects of Bioscience and is a key skill for undergraduate students to develop.

Note: Micropipettors vary by manufacturer, but the most common micropipettes are based on a similar model and work on the principle of air displacement.

How to use micropipettes

1. Familiarise yourself with the micropipette animated activity resources from Learning Science and Support Videos available on NOW (BIOL10272 Practical Techniques & *hyperlink*).
   a) Prepare an Automated Pipette
   b) Operating an Automated Pipette
   c) Accurate Pipetting
   d) Pipette Volumes
2. The micropipettes come in three different sizes which differ in the range that they can accurately dispense liquids
   - 2-20 µl
   - 20-200 µl
   - 200-1000 µl

   The volume that you need to dispense must always be within the range of the micropipette you are using. For example, 1 µl cannot be dispensed from a 2-20 µl micropipette. To dispense a volume of 85 µl, only use the 20-200 µl micropipette and so on. Never rotate the volume of a pipette above or below the range it is designed to dispense. For example, a 2-20 µl micropipette must not be set below 2 µl or above 20 µl..
3. Micropipettes must always be held in upright position. The ejector button has three positions.

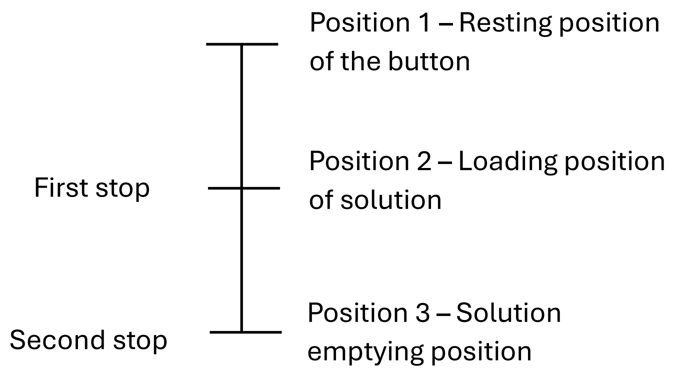

In order to measure the correct liquid volume accurately, these three positions of the ejector button are very important

4. Loading and emptying

Please watch the videos to visually feel how to handle the micropipette.

https://www.youtube.com/watch?v=QGX490kuKjg

https://www.youtube.com/watch?v=ZjGy0cDfkPg&feature=youtu.be

These are very small volumes. Hence the following steps while loading and emptying the sample are very critical.
1. Before starting loading press the ejector button to position 2.
2. Always dip the micropipette tip in the liquid solution just enough to load the required volume (do not touch the micropipette with the liquid).
3. Now transfer the micropipette tip to the eppendorf tube into which liquid needs emptying. Carefully touch the micropipette tip to the eppendorf tube wall and gently press the ejector button until position 3 to remove all the liquid from the micropipette tip.

Some of the practice experiments designed to help you use micropipettes at home.

Before beginning with the experiments please watch this video to understand how to read correct volumes on micropipettes

- Pipette volumes (https://www.youtube.com/watch?v=EEs2UROZsgI&feature=youtu.be)

Exercise 1: The 2,3,5 exercise using a 2-20 µl micropipette
(Watch video - Exercice 1)

Aim of the experiment: To check the accuracy and handling of 2-20 µl micropipette.

Protocol:
1. Make a coloured solution with “Dye Solution Concentrate” supplied in your pack.
2. Pipette 2 µl in a clean microfuge tube. Add another 3 µl of “Dye Solution Concentrate” into the same microfuge tube. Then add a further 5 µl of “Dye Solution Concentrate into the same microfuge tube. The total volume in the microfuge tube should now be 10 µl.
3. Now using a clean tip set the pipette to 10 µl volume and gently remove (aspirate) the total 10 µl of “Dye Solution Concentrate” from the microfuge tube to the fresh pipette tip. You should be able to remove all 10 □l from the tube, leaving nothing behind in the microfuge tube and, importantly, no additional air space at the end of the pipette tip.
4. This exercise requires practice and accuracy to ensure you can reproduce this every time. Repeat this exercise at least 3 times to ensure that you can master this.

Reflection on Exercise 1:

The outcome of this exercise is to practice handling small volumes accurately using micropipette.

Note: If there is any pipetting error in creating the final volume of 10 µl then this will result in incorrect amounts left in the microfuge tube (if the total volume is more that 10 µl) or an air bubble in the tip (if the total volume is less than 10 µl). There is also the possibility of error when attempting to aspirate the 10 µl volume..

Exercise 2: The 2,3,5 exercise using a 20-200 µl or a 200-1000 µl pipettes.

(Watch video - Exercice 2)

Protocol: Following the same protocol as above use the following set of volumes.

Using the 20-200 µl pipette

- Add 23 µl, 127 µl and 50 µl and aspirate 200 µl
- Add 145 µl, 26 µl and 29 µl and aspirate 200 µl
- Add 20 µl, 10 times and take out 200 µl

Using the 200-1000 µl pipette

- Add 225 µl, 445 µl and 330 µl and aspirate 1000 µl.
- Add 334 µl, 242 µl and 424 µl and aspirate 1000 µl.
- Add 200 µl, 5 times and take out 1000 µl.

Repeat the exercise at least 3 times for each of the micropipettes to ensure that you can master this.

Exercise 3: Making dilutions

(Watch video - Exercice 3)

Please watch this video to understand the dilution technique

- How to dilute solutions (https://www.youtube.com/watch?v=41hBBw56Ac4&feature=youtu.be)

Aim: Preparing small and accurate concentrations of diluted solutions .

I. Protocol:

1. Use "Dye Solution Concentrate" solution as stock solution.
2. In order to make dilutions from this stock solution use water as diluent (in which you will be adding stock solution)

3. Make a 1 in 2 dilution, follow the guide below.
   Take clean microfuge tube. Remember to label the tube with the final volume.
   - Take out 10 µl of the "Dye Solution Concentrate" solution in a microfuge tube and add 10 µl of water. The total volume will be 20 µl.
   - Take 100 µl of the "Dye Solution Concentrate" solution in a microfuge tube and add 100 µl of water. The total volume will be 200 µl.
   - Take 20 µl of the "Dye Solution Concentrate" solution in a microfuge tube and add 20 µl of water. The total volume will be 40 µl.
   - Take 50 µl of the "Dye Solution Concentrate" solution stock solution in a microfuge tube and add 50 µl of water. The total volume will be 100 µl.

Results:

Take a picture of all the tubes in which dilutions are made. Notice the colour should be same in all the tubes despite the fact that the volumes are different.

II. Repeat the above protocol for 1 in 5 dilution and 1 in 10 dilution.

4. To make 1 in 5 dilution, follow the guide below. Remember to label the tubes with final volume.
   - Take out 10 µl of the "Dye Solution Concentrate" solution in a microfuge tube and add 40 µl of water. The total volume will be 50 µl.
   - Take out 20 µl of the "Dye Solution Concentrate" solution in a microfuge tube and add 80 µl of water. The total volume will be 100 µl.
   - Take out 100 µl of Dye Solution Concentrate" solution in a microfuge tube and add 400 µl water. The total volume will be 500 µl.

Results:

Take a picture of the tubes and notice the colour change and volumes.

5. To make 1 in 10 dilution, follow the guide below. Remember to label the tubes with final volume.

   - Take 10 µl of "Dye Solution Concentrate" solution in a microfuge tube into a clean microfuge tube and add 90 µl of water. The total volume will be 100 µl.
   - Take 2 µl of Dye Solution Concentrate" solution in a microfuge and add 18 µl of water. The total volume will be 20 µl.
   - Take 50 µl of Dye Solution Concentrate" solution in a microfuge and add 450 µl of water. The total volume will be 500 µl.
   - Take 100 µl of "Dye Solution Concentrate" solution in a microfuge and add 900 µl of water. The total volume will be 1000 µl.

Results:

Take a picture of the tubes and notice the colour change and volumes.

<u>Exercise 4:</u> Accuracy versus Precision

(Watch video - Exercice 4)

Aim: This experiment is designed to measure a given volume accurately using a micropipette by adding a drop of coloured solution on to a filter paper (provided in the pack).

Protocol:

1. Use "Dye Solution Concentrate" solution.
2. Prepare a thick sheet of filter paper. To avoid leakage and spillages. Ensure you are working in a suitable wipe down area or alternatively place a few layers of paper towels underneath and around your work area.
3. Mark the areas where you will be adding drops following the template shown below as an example.

    Top left to right markings 1 cm apart.

    Left panel from top to bottom 2 cm apart.

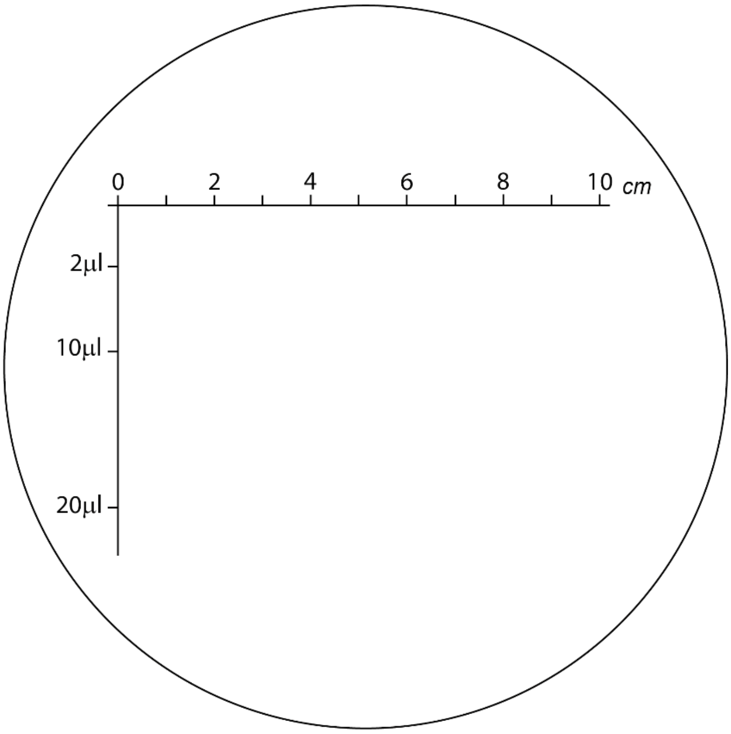

4. Using 2-20 µl, add 10 individual drops of either 2 µl, 10 µl and 20 µl under the 1 cm markings. Progress towards right after completing each liquid dispensation.
5. Obtain3 replicates of data by collecting 2 more data sets from fellow students in your tutorial group.

Results: Using the either a ruler or MS Word, measure the diameters of each row of drops. You will have 10 readings for each set of dispensed volume.

6. Now using another piece of filter paper and following the above steps, use the20-200 µl pipette and add 20 µl, 50 µl, 100 µl and 200 µl.

   Note: As the volumes get bigger the dots will need to be placed further apart from each other (more than 1 cm apart).

Results: Using the either a ruler or MS Word, measure the diameters of each row of drops. You will have 10 readings for each set of dispensed volume.

Data representation:

In a table collect all the measurements for diameters in cm:

Table 1. Pipetting accuracy. Diameter measurements of dispensed volumes.

S.No	2-20 µl pipette			20-200 µl pipette			
	2 µl	10 µl	20 µl	20 µl	50 µl	100 µl	200 µl
1							
2							
3							
4							
5							
6							
7							
8							
9							
10							

This table represents one data set. To check the reproducibility 2 more data sets can be collected from fellow students in your tutorial group.

Statistical analysis:

In your workshop session, you will explore how to perform statistical analysis to analyse the data, which can be plotted in a graph with error bars. One of the statistical tests you could perform is ANOVA. This link demonstrates the ANOVA test

https://www.youtube.com/watch?v=nmHFFFpOVZs.

How to set up microscope at home

Using a smartphone (recommended)

1. Unpack your microscope from the box and use the cable to charge it. You will need an adapter from USB to a 3 pin UK plug - you may already have one for your mobile phone or another device. <u>Make sure you plug the cable into a power socket, not a computer, for charging</u>. Check that the small red LED light on the top of the microscope is on and allow it to charge for 3-4 hours.

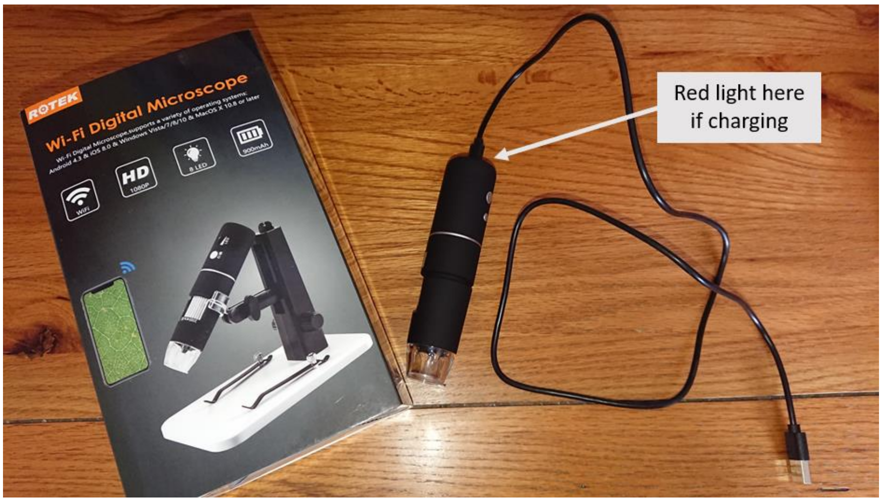

2. Find the Max-see app in Google Play or Apple store and install it on your smartphone.

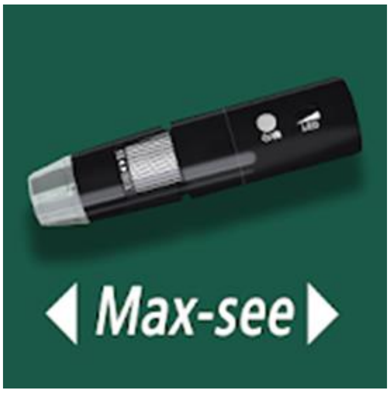

3. Remove the protective cover and switch on the microscope by pressing and holding the on/off button until the white light at the bottom of the microscope turns on.

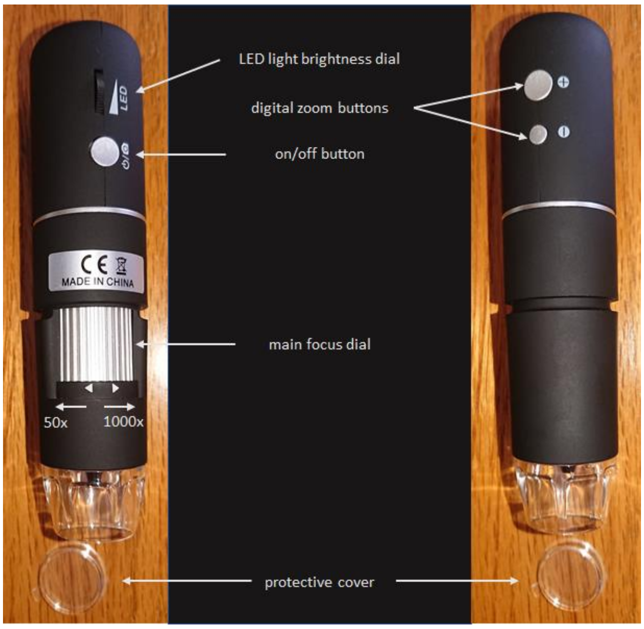

4. Go to the wifi settings on your mobile phone and connect to Max-See wifi. This will connect the microscope to your phone, and you should then be able to use the microscope like a camera, via the Max-See app.

5. Open the Max-See app.

6. Place the microscope in its stand and try to focus on a simple object. For example, you could choose a piece of paper or fabric. The end of the microscope must be close to the object for best results.

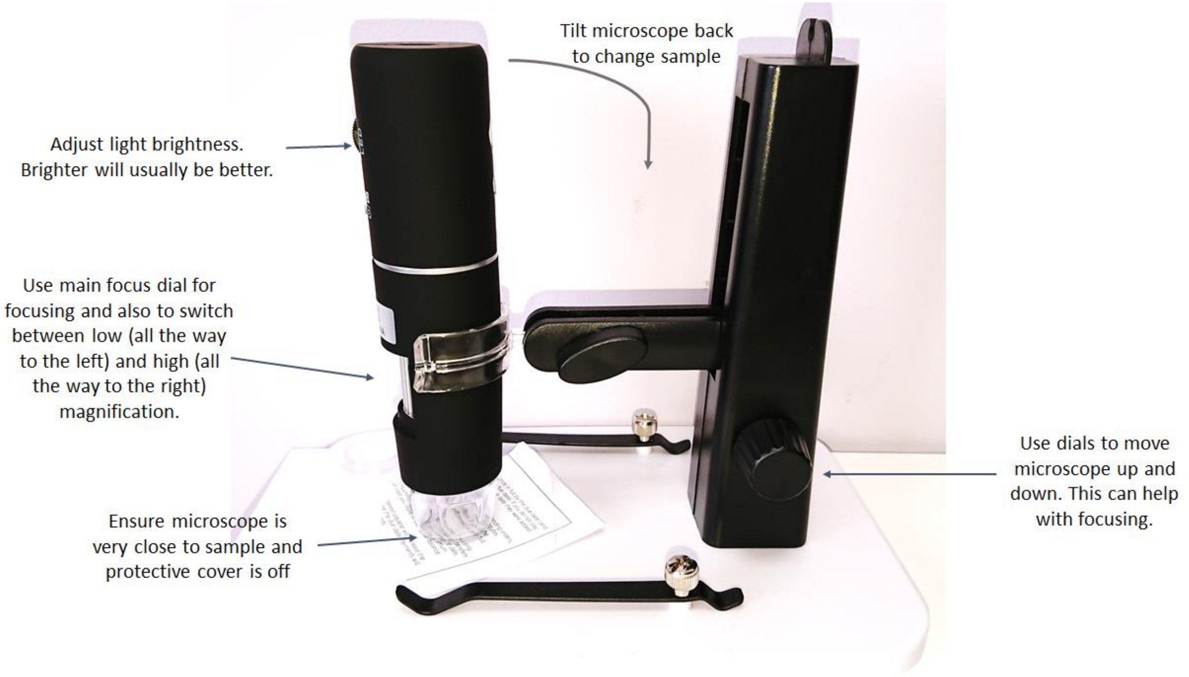

7. Once you have focused your image, try taking a photo or short video using the Max-See app.

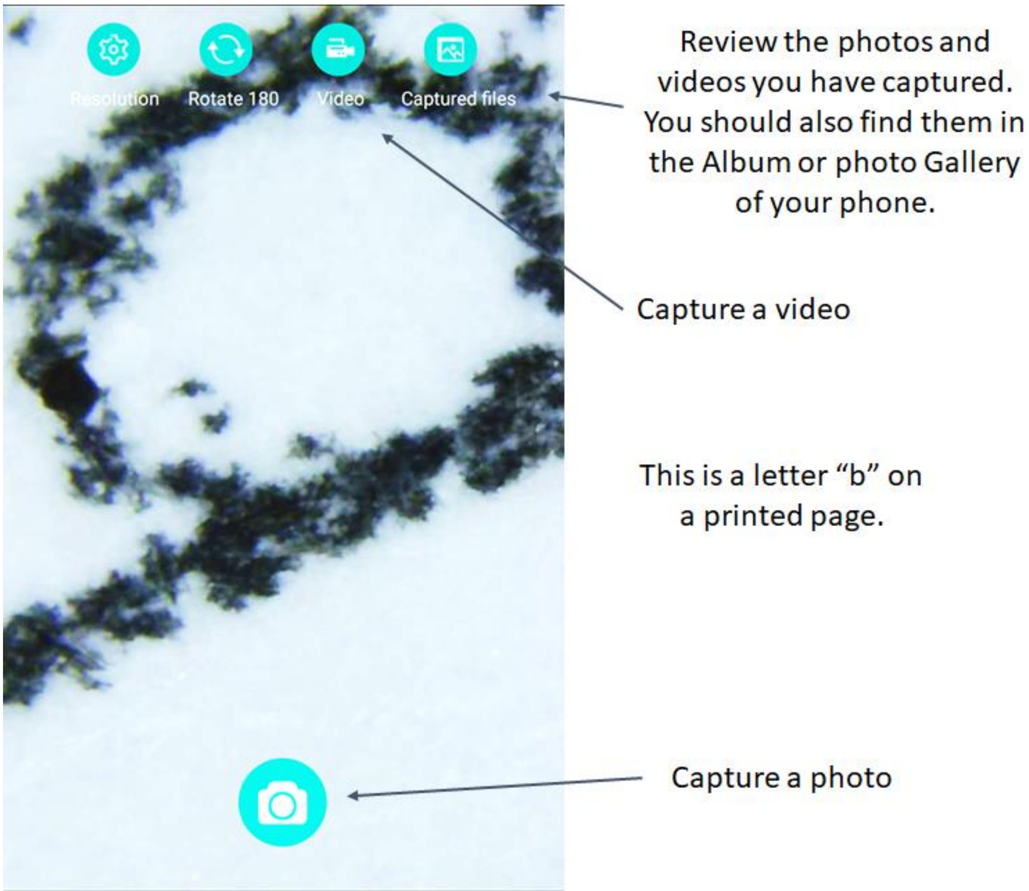

Using a computer or laptop

It is also possible to connect your microscope to a computer, though we have found the smartphone method most straightforward for most users.

Your microscope should be compatible with computers running Windows 7/8/10 and Mac 10.8 or higher.

To connect your microscope to a computer, first charge the microscope (using a power socket, *not* a computer USB port).

Install the appropriate software on your computer, then connect the microscope to your computer using the USB cable. Then follow the instructions below.

Installation for a Mac

1. Press and hold the on/off button on the microscope to turn it on.

2. Open the "photo booth" app on your Mac.

3. Select the camera option "General-UVC#2 (default).

Installation for a Windows computer

1. Download the smart camera software from www.inskam.com/download/camera.zip and run the application that is inside the downloaded zip folder.

2. Press and hold the on/off button on the microscope to turn it on.

3. Allow your computer to install the USB Driver. If the installation completes successfully, proceed to step 4. If not, try a different USB port on your computer or remove then plug the device in again.

4. Make sure the Settings tab has the Device listed as "GENERAL-UVC". If not, then change it using the Device dropdown menu on the top left. You can change the location of saved photos and videos using the "Path" settings on the right.

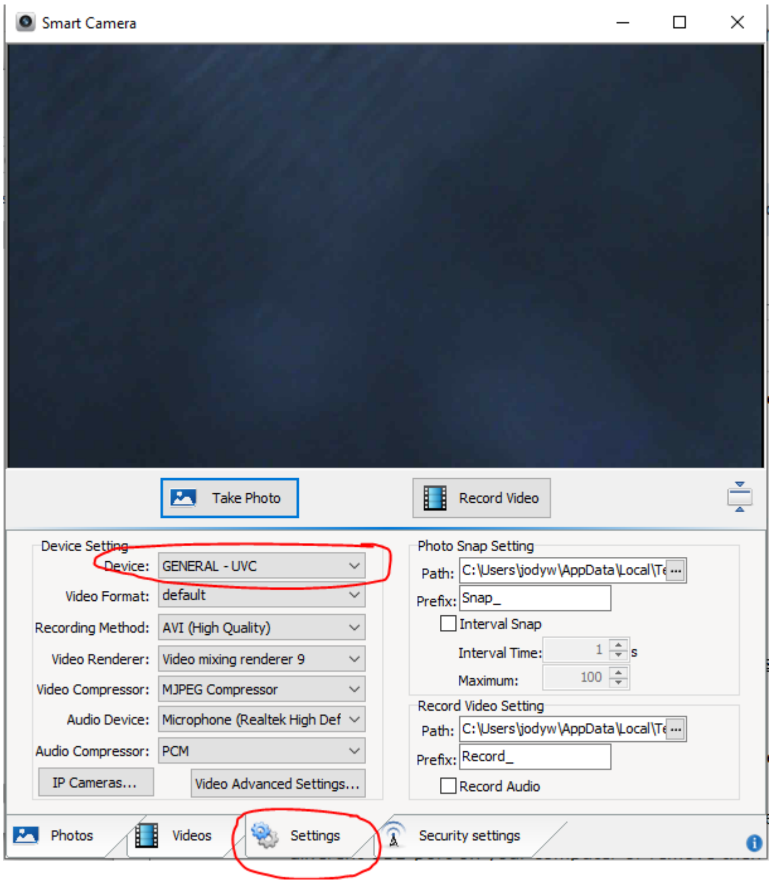

Haemocytometer as a scale

In your bioskills @home learning pack you have two haemocytometer slides:

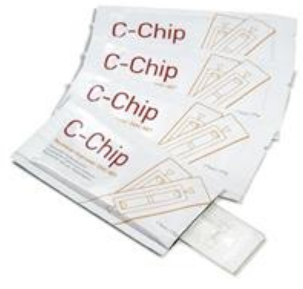

You can use one of them to estimate the size of microscopic items if you wish.

The haemocytometer grid will be most visible over a smooth, black background - we found that a kitchen bin liner worked quite well:

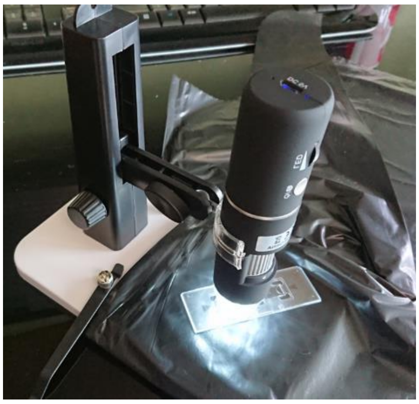

The grid you will see includes squares of different sizes. Below the dimensions of a small square are shown for reference:

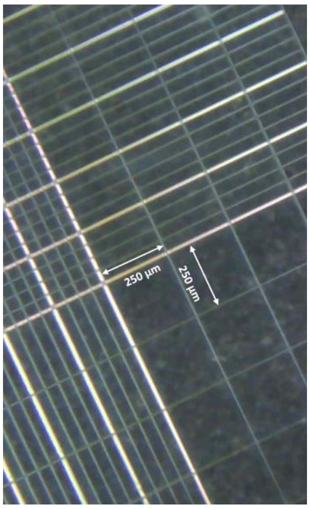

Supplement: Supplementary Material 2. [file acmi-8-01157-s002.pdf]
